# Genome-Wide Association Study of Lp-PLA_2_ Activity and Mass in the Framingham Heart Study

**DOI:** 10.1371/journal.pgen.1000928

**Published:** 2010-04-29

**Authors:** Sunil Suchindran, David Rivedal, John R. Guyton, Tom Milledge, Xiaoyi Gao, Ashlee Benjamin, Jennifer Rowell, Geoffrey S. Ginsburg, Jeanette J. McCarthy

**Affiliations:** 1 Institute for Genome Sciences and Policy, Duke University Medical Center, Durham, North Carolina, United States of America; 2 Bioinformatics Research Center, North Carolina State University, Raleigh, North Carolina, United States of America; 3 Department of Medicine, Division of Endocrinology, Metabolism, and Nutrition, Duke University Medical Center, Durham, North Carolina, United States of America; 4 Scalable Computing Support Center, Duke University Medical Center, Durham, North Carolina, United States of America; 5 Division of Statistical Genomics, Washington University School of Medicine, St. Louis, Missouri, United States of America; 6 Department of Community and Family Medicine, Duke University Medical Center, Durham, North Carolina, United States of America; University of Oxford, United Kingdom

## Abstract

Lipoprotein-associated phospholipase A_2_ (Lp-PLA_2_) is an emerging risk factor and therapeutic target for cardiovascular disease. The activity and mass of this enzyme are heritable traits, but major genetic determinants have not been explored in a systematic, genome-wide fashion. We carried out a genome-wide association study of Lp-PLA_2_ activity and mass in 6,668 Caucasian subjects from the population-based Framingham Heart Study. Clinical data and genotypes from the Affymetrix 550K SNP array were obtained from the open-access Framingham SHARe project. Each polymorphism that passed quality control was tested for associations with Lp-PLA_2_ activity and mass using linear mixed models implemented in the R statistical package, accounting for familial correlations, and controlling for age, sex, smoking, lipid-lowering-medication use, and cohort. For Lp-PLA_2_ activity, polymorphisms at four independent loci reached genome-wide significance, including the *APOE/APOC1* region on chromosome 19 (p = 6×10^−24^); *CELSR2/PSRC1* on chromosome 1 (p = 3×10^−15^); *SCARB1* on chromosome 12 (p = 1×10^−8^) and *ZNF259/BUD13* in the *APOA5/APOA1* gene region on chromosome 11 (p = 4×10^−8^). All of these remained significant after accounting for associations with LDL cholesterol, HDL cholesterol, or triglycerides. For Lp-PLA_2_ mass, 12 SNPs achieved genome-wide significance, all clustering in a region on chromosome 6p12.3 near the *PLA2G7* gene. Our analyses demonstrate that genetic polymorphisms may contribute to inter-individual variation in Lp-PLA_2_ activity and mass.

## Author Summary

Blood levels of lipoprotein-associated phospholipase A_2_ (Lp-PLA_2_) show a strong association with atherosclerosis in humans. This enzyme is made by certain cells of the immune system, associates with lipoproteins (HDL and LDL), and is thought to be involved in inflammation. Studies have shown that Lp-PLA_2_ is a good predictor of cardiovascular disease, independent of HDL and LDL cholesterol levels. This has led to the development of drugs aimed at inhibiting Lp-PLA_2_ as a way to treat or prevent cardiovascular disease. The activity and mass of Lp-PLA_2_ are heritable traits, but major genetic determinants have not been explored in a systematic fashion. We examined genetic variants across the human genome to identify genes influencing Lp-PLA_2_ activity and mass. We studied 6,668 Caucasian subjects from the population-based Framingham Heart Study. Clinical data and genetic data on 550,000 genetic variants were available for association analysis. There was no overlap in the most significantly associated SNPs for activity and mass. We identified four distinct gene regions showing highly significant associations with Lp-PLA_2_ activity, all of which are known to include genes involved in cholesterol metabolism. The only locus associated with Lp-PLA_2_ mass was a region harboring *PLA2G7*, the gene that encodes lipoprotein-associated phospholipase A2.

## Introduction

It is increasingly recognized that inflammation plays an important role in the development of atherosclerotic cardiovascular disease. Among the most studied circulating biomarkers of inflammation in this setting is lipoprotein-associated phospholipase A_2_ (Lp-PLA_2_). Studies in animal models with human-like lipoprotein metabolism have suggested that Lp-PLA_2_ activity contributes to atherosclerosis development, and epidemiological studies have presented evidence that circulating concentrations of Lp-PLA_2_ or elevated activity of the enzyme are associated with cardiovascular disease after adjusting for established risk factors [1]–[3]. This has led to the recent development of an orally active Lp-PLA_2_ inhibitor currently in clinical trials for cardiovascular prevention [3].

Lp-PLA_2_, also known as platelet-activating factor acetylhydrolase (PAF-AH), is produced by cells of hematopoietic lineage (monocytes/macrophages, T-lymphocytes, mast cells, megakaryocytes and platelets). The enzyme reacts with oxidized phospholipids to generate the pro-inflammatory lipids lysophosphatidylcholine and oxidized free fatty acids. The relationship between Lp-PLA_2_ and atherosclerosis is complex. Lp-PLA_2_ is non-covalently bound to human plasma lipoproteins; the binding site on the Lp-PLA_2_ molecule differs according to whether the carrier lipoprotein is LDL or HDL [4]. Most Lp-PLA_2_ is bound to LDL, and subjects with elevated plasma LDL levels also have high Lp-PLA_2_ activity [5]. In epidemiologic studies, Lp-PLA_2_ activity and mass reflect predominantly LDL-associated Lp-PLA_2_ [6]. Importantly, Lp-PLA_2_ is highly associated with the smallest LDL and HDL subclasses [7] and with electronegative LDL, which overlaps with small dense LDL [8]. Lp-PLA_2_ bound to HDL has a much lower specific activity than that bound to LDL [6]. Despite the fact that HDL carries some of the Lp-PLA_2_ activity in plasma, we and others have found an inverse relationship between Lp-PLA_2_ activity and HDL levels [9]. This is likely explained by the fact that small dense LDL (with high Lp-PLA_2_ activity) are more abundant in subjects with lower levels of HDL. Small dense LDL are also found in the metabolic syndrome, which is associated with high Lp-PLA_2_ activity [10].

Given the importance of Lp-PLA_2_ as a biomarker of cardiovascular disease pathogenesis and the development of therapeutics directed at Lp-PLA_2_, a better understanding of how genetic variation controls Lp-PLA_2_ activity and mass is warranted. Pursuant to this goal, Schnabel *et al.* estimated the heritabilities of Lp-PLA_2_ activity and mass to be 0.41 and 0.25, respectively, in the Framingham Heart Study, which is consistent with estimates from other cohorts [11], [12]. They also conducted a large-scale analysis of candidate genes, focusing on variants in *PLA2G7*, the gene that encodes Lp-PLA_2_, and other SNPs in inflammatory genes [11]. Their study identified variants of potential interest, but they noted that no SNPs achieved experiment-wide statistical significance. In the present study, we conducted a genome-wide association study (GWAS) to identify additional candidate genes that emerge on a genome-wide scale. Using genotype and clinical data available on participants in the Framingham Heart Study, we carried out an analysis of common genetic variants across the genome to identify loci associated with Lp-PLA_2_ activity and mass. For Lp-PLA_2_ activity, our study revealed four genomic regions reaching genome-wide significance, all harboring genes involved in lipid metabolism. For Lp-PLA_2_ mass, the only associated locus was a region harboring *PLA2G7*, the gene that encodes Lp-PLA_2_.

## Results

**Figure 1:**
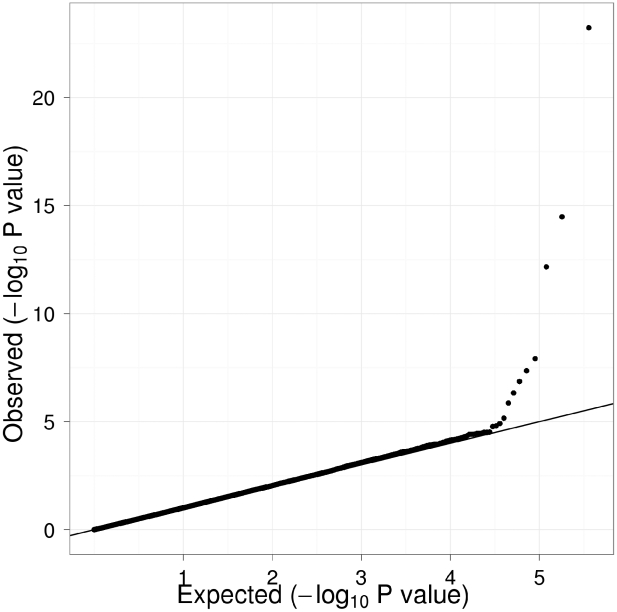
Q-Q plot of genome-wide P values for association with Lp-PLA_2_ activity. Regression analysis of Lp-PLA_2_ activity in Caucasians from the Framingham Heart Study. Model controlled for age, age^2^, sex, lipid-lowering medication use, cohort, and smoking status.

**Figure 2:**
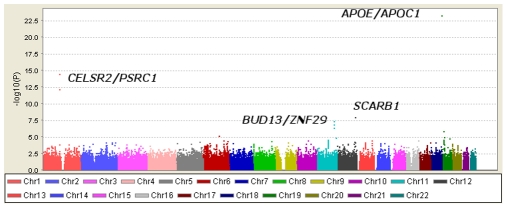
Manhattan plot of genome-wide P values for association with Lp-PLA_2_ activity. Polymorphisms in four regions reached genome-wide significance and are labeled with candidate genes in the associated region.

**Figure 3:**
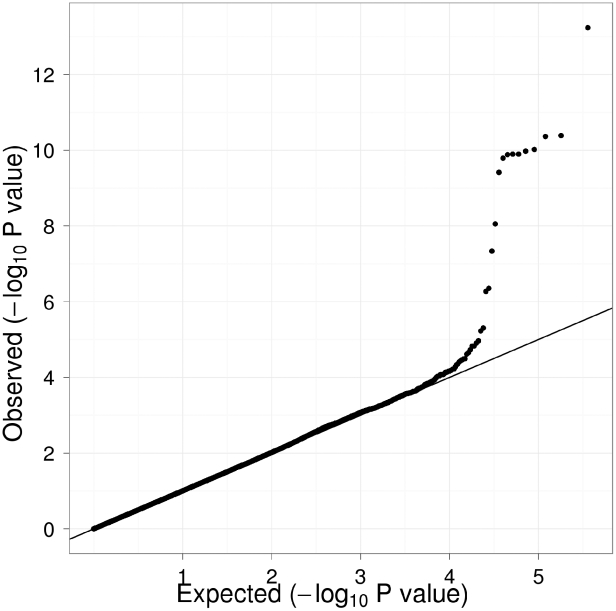
Q-Q plot of genome-wide P values for association with Lp-PLA_2_ mass. Regression analysis of Lp-PLA_2_ activity in Caucasians from the Framingham Heart Study. Model controlled for age, age^2^, sex, lipid lowering medication use, cohort, and smoking status.

**Figure 4:**
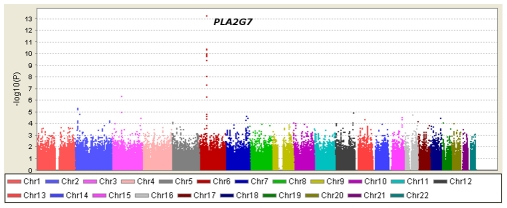
Manhattan plot of genome-wide P values for association with Lp-PLA_2_ mass. Only the region around the *PLA2G7* gene reached genome-wide significance.

Table 1 displays clinical characteristics of the population under study. The Third Generation cohort is younger and has a more favorable lipid profile as compared to the Offspring cohort. It also has higher Lp-PLA_2_ activity, but the variance within each cohort is similar. Graphical summaries of the genome-wide associations with Lp-PLA_2_ activity (Figure 1 and Figure 2) and mass (Figure 3 and Figure 4) are displayed using a quantile-quantile (Q-Q) plot and a Manhattan plot. The Q-Q plot for each phenotype reveals a clear departure from the overall-null hypothesis of no associations. The corresponding Manhattan plots present an alternative view showing the separation of top hits among the genome-wide associations. The test statistics that generated these summaries do not display evidence of inflation (inflation factors = 1.007 and 0.998) and hence, we did not adjust for population structure. Results for all SNPs tested can be found in Table S1 (activity) and Table S2 (mass).

**Table 1:** Descriptive statistics from the Framingham Heart Study population used in the current study.

	OffspringExam 7	Generation 3Exam 1	Combined
Sample Size	N = 3023	N = 3645	N = 6668
Year of enrollment	1998–2001	2002–2005	
Female, N (%)	1609 (53)	1938 (53)	3547 (53)
Smoke Regularly, N (%)	409 (14)	637 (17)	1046 (16)
Diabetes, N (%)	335 (11)	107 (3)	442 (7)
Lipid lowering therapy, N (%)	639 (21)	315 (9)	954 (14)

	*Mean (SD)*	*Mean (SD)*	*Mean (SD)*
Age in years	61 (9)	40 (9)	48 (14)
Lp-PLA_2_ activity, nmol/ml/min	143(36)	166 (36)	155 (38)
Lp-PLA_2_ mass, ng/mL	299 (94)	231 (48)	263 (81)
LDL-C, mg/dL	120(33)	112 (32)	116 (32)
HDL-C, mg/dL	54 (17)	54 (16)	54 (17)
Total cholesterol, mg/dL	200 (37)	189 (36)	194 (37)
Triglycerides, mg/dL *	116(88)	92 (75)	103 (81)
Body Mass Index, kg/m^2^	28 (5)	27 (6)	27 (5)

Data presented are calculated ignoring familial clustering.

*median and interquartile range.

### Loci achieving genome-wide significance for association with Lp-PLA_2_ activity

**Figure 5:**
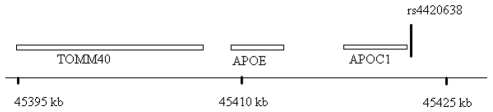
Genomic region surrounding the top hit near *APOE/APOC1*. Location of rs4420638 is shown relative to genes in the region of chromosome 19q13.

Six SNPs in four distinct loci were associated with Lp-PLA_2_ activity at a genome-wide level of significance in a model controlling for age, age^2^, sex, cohort, lipid-lowering-medication use, and smoking (Table 2). The top hit was the SNP ss66185226 (rs41377151), which is identical to the SNP rs4420638. This SNP is located approximately 340 bp from the 3′ end of the apolipoprotein C-I (*APOC1*) gene within the *APOE/APOC1* gene cluster on chromosome 19q13.32 (Figure 5). Two other SNPs – rs599839, located 10 bp downstream of *PSRC1* and rs4970834, located in an intron of *CELSR2* – are in moderate linkage disequilibrium (r^2^ = 0.63) with each other and represent a second locus that maps to chromosome 1p13.3, which includes the genes *PSRC1*, *CELSR2*, and *SORT1*. The fourth hit, rs10846744, lies within an intron of *SCARB1* on chromosome 12q24.31. The final two hits, rs12286037 and rs11820589, are in high linkage disequilibrium (r^2^ = 0.85), and map to 11q23.3. rs12286037 lies within an intron of the gene *ZNF259*, and rs11820589 resides within the *BUD13* gene. Both SNPs are nearby an apolipoprotein gene cluster containing *APOA5*, *APOA4*, *APOC3* and *APOA1*. All tested SNPs and their p-values can be found in Table S1.

**Table 2:** Association (beta estimates and p values) of top activity and mass SNPs with Lp-PLA_2_ activity, mass, lipoproteins, and apolipoproteins.

	*APOC1*	*PSRC1*	*SCARB1*	*ZNF259*	*PLA2G7*
Top SNP	rs4420638	rs599839	rs10846744	rs12286037	rs1805017
Distance to gene (bp)	340	10	0	0	0
Minor allele (frequency)	G (0.16)	G (0.22)	C (0.15)	T (0.07)	T (0.26)

		*Regression coefficient (p value)*			
Lp-PLA2 activity	8.0 (5.9×10^−24^)	−5.5 (3.3×10^−15^)	4.6 (1.2×10^−8^)	6.4 (4.4×10^−8^)	−2.1 (1.5×10^−3^)
Lp-PLA2 mass	ns	−4.6 (3.4×10^−3^)	7.8 (1.4×10^−5^)	ns	11.1 (5.8×10^−14^)
LDL-C	5.2 (3.3×10^−11^)	−5.7 (1.8×10^−16^)	ns	ns	ns
HDL-C	−1.1 (3.3×10^−3^)	ns	ns	−2.2 (7.3×10^−5^)	ns
Triglycerides	ns	ns	ns	1.1 (4.0×10^−11^)	ns
Apolipoprotein B	4.3 (5.6×10^−7^)	−4.7 (4.8×10^−10^)	ns	4.3 (4.6×10^−4^)	ns
Apolipoprotein A1	ns	ns	ns	ns	ns
Apolipoprotein E	−2.1 (8.1×10^−31^)	ns	ns	ns	ns

Apolipoproteins A and B were measured at Exam 4 and Apolipoprotein E measured at Exam 5 of the Offspring cohort. Therefore, the sample size (n = 3144) differs from the sample size used for SNP associations with Lp-PLA_2_ and lipoproteins, which were measured in both the Offspring cohort and the third generation cohort(n = 6668). All models controlled for age, age2, cohort, sex, lipid-lowering medication use, and smoking. P-values greater than 0.01 indicated as not significant (ns).

In considering how much phenotypic variation can be explained by the top hits, the mixed model presents some challenges. A measure equivalent to R^2^ does not exist because phenotypic variation is partitioned into two variance components: an additive polygenic component and residual error. To estimate the impact that the top activity loci had on explaining the variance of this trait, we compared models with and without our top SNPs and found that adding the associated SNPs decreased the polygenic variance by 4.7% and the residual variance by 3.1%.

### Loci achieving genome-wide significance for association with Lp-PLA_2_ mass

**Figure 6:**
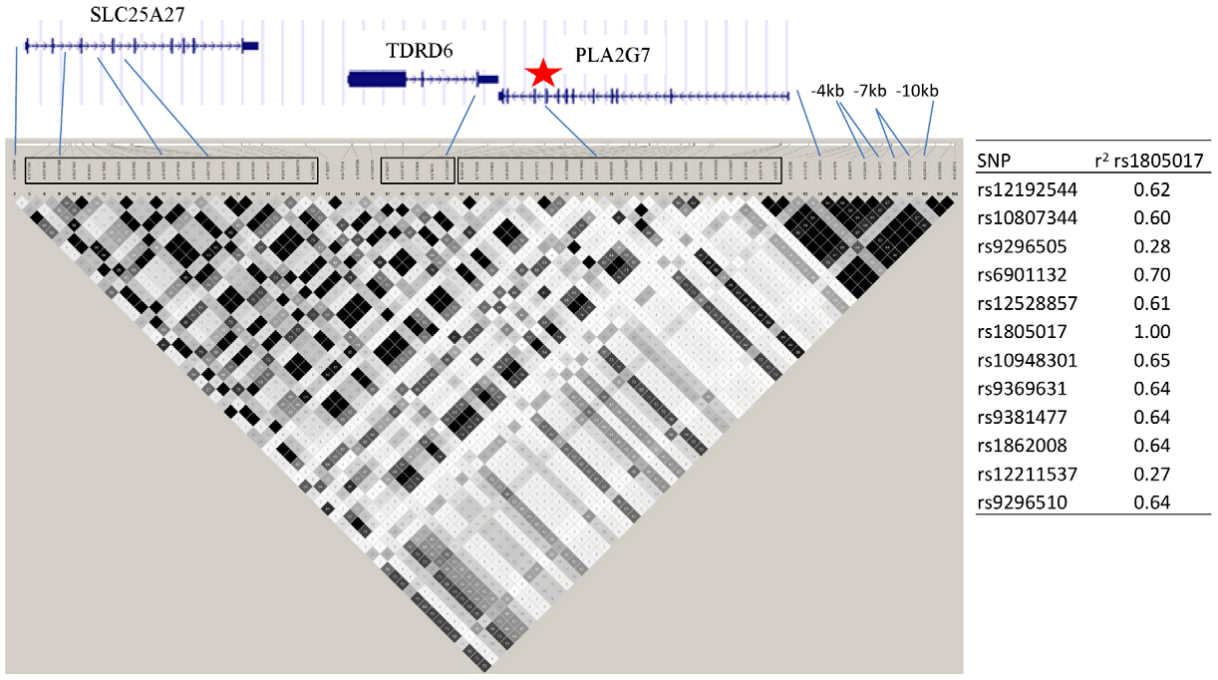
*PLA2G7* gene and surrounding region. Linkage disequilibrium (r^2^) plot across the *PLA2G7* gene region, including all SNPs significantly associated with Lp-PLA_2_ mass (listed). Figure was generated from HapMap Caucasian data using Haploview software. Star indicates location of the top SNP, rs1805017, in exon 4 of the *PLA2G7* gene. Table to the right of the figure shows linkage disequilibrium (r^2^) between all of the SNPs associated with Lp-PLA_2_ mass and the top hit, rs1805017, generated from Framingham data.

Twelve SNPs clustered in a region of chromosome 6p12.3 were associated with Lp-PLA_2_ mass at a genome-wide level of significance (Figure 6). The top hit, rs1805017, is a non-synonymous change (H92R) within the *PLA2G7* gene, encoding lipoprotein-associated phospholipase A2. The T allele (corresponding to the amino acid histidine) was associated with higher Lp-PLA_2_ mass. A closer evaluation of this region suggests that linkage disequilibrium with the top SNP, rs1805017, is driving the remaining associations in this region.

### Analyses of Lp-PLA_2_ activity, conditioning on mass, and vice versa

We observed that the top hits for Lp-PLA_2_ activity and mass did not overlap. The correlation between activity and mass in the sample (ignoring familial clustering) was 0.27, so these phenotypes are not expected to carry the same information. However, the top mass SNP, rs1805017, was weakly associated with Lp-PLA_2_ activity (p = 1.5×10^−3^), so we investigated whether the activity association could be explained by this SNP's association with Lp-PLA_2_ mass by including mass as a covariate. Instead, including Lp-PLA_2_ mass in the model considerably strengthened the association of this SNP with activity (Table 3, p = 2.1×10^−12^).

**Table 3:** The effect of controlling for activity in analyses of Lp-PLA_2_ mass and vice versa on SNP associations, presented as regression coefficient (p value).

SNP	Closest Gene	Activity	Mass-adjusted Activity	Mass	Activity-adjusted Mass
rs4420638	*APOC1*	8.0 (5.9×10^−24^)	7.9 (1.6×10^−28^)	ns	−7.5(2.5×10^−6^)
rs599839	*PSRC1*	−5.5 (3.3×10^−15^)	−4.7 (1.4×10^−13^)	−4.6 (3.4×10^−3^)	ns
rs4970834	*CELSR2*	−5.5 (6.8×10^−13^)	−4.7 (1.6×10^−11^)	−4.6 (6.7×10^−3^)	ns
rs10846744	*SCARB1*	4.6 (1.2×10^−8^)	3.2 (1.1×10^−5^)	7.8 (1.4×10^−5^)	ns
rs12286037	*ZNF259*	6.4 (4.4×10^−8^)	5.5 (1.5×10^−7^)	ns	ns
rs11820589	*BUD13*	6.2 (1.4×10^−7^)	5.5 (3.1×10^−7^)	ns	ns
rs1805017	*PLA2G7*	−2.1 (1.5×10^−3^)	−4.2 (2.1×10^−12^)	11.1 (5.8×10^−14^)	13.3(4.3×10^−24^)
rs12192544	*SLC25A27*	ns	−3.0 (2.2×10^−6^)	10.3 (4.7×10^−11^)	11.4 (2.0×10^−16^)
rs12528857	*TDRD6*	ns	−3.0 (1.3×10^−6^)	10.3 (5.0×10^−11^)	11.5 (1.5×10^−16^)
rs9369631	*PLA2G7*	ns	−3.4 (1.7×10^−7^)	10.3 (9.5×10^−11^)	11.7 (1.3×10^−16^)
rs1862008	*PLA2G7*	ns	−3.4 (2.1×10^−7^)	10.3 (1.1×10^−10^)	11.7 (1.6×10^−16^)
rs10948301	*PLA2G7*	ns	−3.3 (2.4×10^−7^)	10.2 (1.3×10^−10^)	11.7 (2.0×10^−16^)
rs9296510	*PLA2G7*	ns	−3.4 (2.1×10^−7^)	10.2 (1.3×10^−10^)	11.7 (2.0×10^−16^)
rs10807344	*SLC25A27*	ns	−2.9 (3.4×10^−6^)	10.0 (1.5×10^−10^)	11.1 (1.0×10^−15^)
rs9381477	*PLA2G7*	ns	−3.4 (2.4×10^−7^)	10.2 (1.6×10^−10^)	11.6 (2.8×10^−16^)
rs9296505	SLC25A27	ns	ns	−8.2 (3.7×10^−10^)	−8.0 (7.3×10^−12^)
rs12211537	*PLA2G7*	ns	ns	−7.5 (9.2×10^−9^)	−7.4 (1.8×10^−10^)
rs6901132	SLC25A27	−1.9 (2.7×10^−3^)	−3.3 (4.6×10^−9^)	7.5 (4.8×10^−8^)	9.3 (2.5×10^−14^)
rs1421378	*PLA2G7*	ns	−2.6 (9.7×10^−7^)	ns	8.0 (1.0×10^−11^)
rs6458511	CYP39A1	ns	−2.3 (6.2×10^−5^)	ns	7.2 (8.4×10^−9^)

*All models control for age, age2, cohort, sex, lipid-lowering medication use, and smoking. P-values greater than 0.01 indicated as not significant (ns).

Because we observed this notable shift in p-values, we repeated our genome-wide association analyses, modeling SNP associations with Lp-PLA_2_ activity while controlling for Lp-PLA_2_ mass, and vice versa (Table 3). For Lp-PLA_2_ mass, the top hits in the *PLA2G7* gene region remained the same, but all p-values became more significant. In addition, two other SNPs in this region, rs1421378 (p = 1×10^−11^) and rs6458511 (p = 8×10^−9^), became significant. We also noted that SNPs near the *APOB* gene at 2p24.1 now approached genome-wide significance (p∼4×10^−7^) for their association with mass.

For analysis of Lp-PLA_2_ activity, controlling for mass uncovered significant associations with two SNPs in linkage disequilibrium with each other (r^2^ = 0.70) at the *PLA2G7* locus: rs1805017(estimated effect: −4.2, p = 1×10^−12^) and rs6901132 (p = 5×10^−9^). The T allele of rs1805017 (histidine), associated with increased mass in our analysis, was associated with decreased activity. Controlling for mass also strengthened the association at the *APOE/APOC1* locus (p = 1.5×10^−28^) and weakened the association at the *SCARB1* locus (p = 1.5×10^−5^). We quantified the amount of variance in activity explained by the rs1805017 SNP along with the SNPs previously associated with activity, in a model that included mass as well. As before, we compared models with and without the genetic variants and found that adding these six SNPs decreased the polygenic variance by 9.4% and the residual variance by 1.7%. For Lp-PLA_2_ mass, including rs1805017 decreases the polygenic variance by 12.2% and increases the residual variance by 0.28%.

### Relationship between top Lp-PLA_2_ activity loci and serum lipid/lipoprotein levels

**Figure 7:**
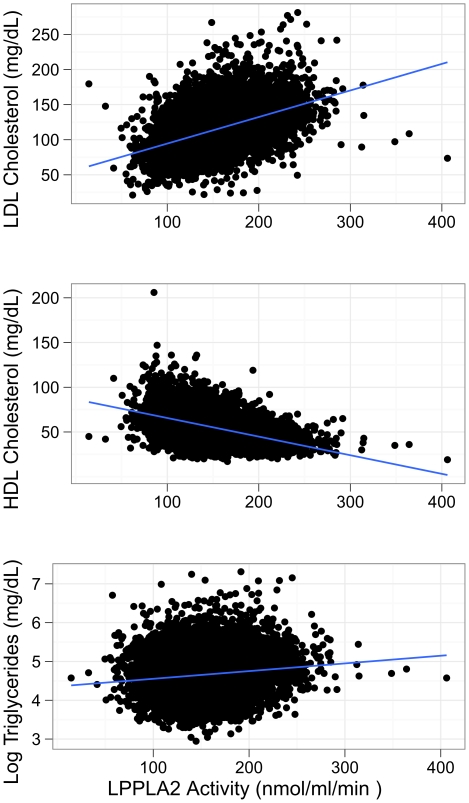
Scatter plot of Lp-PLA_2_ activity against LDL cholesterol, HDL cholesterol, and triglycerides. Units are mg/dL for all lipids/lipoproteins and nmol/ml/min for Lp-PLA_2_ activity. Triglycerides are log-transformed.

All four loci associated with Lp-PLA_2_ activity have generated strong signals in previous association studies for lipid phenotypes and include genes with established roles in lipid metabolism. Therefore, interpreting the results requires understanding the relationship between Lp-PLA_2_ activity and lipid/lipoprotein levels. Figure 7 displays scatter plots (ignoring familial clustering) of Lp-PLA_2_ activity with LDL-C (r = 0.44), HDL-C (r = −0.48), and triglycerides (log-transformed, r = 0.13). The linear trend and moderate sample correlations with LDL-C and HDL-C imply that markers associated with these lipoproteins may also be associated with Lp-PLA_2_ activity to some extent. Indeed, both the *APOE/APOC1* locus and the *CELSR2/PSRC1* locus have demonstrated strong signals in genome-wide association studies of LDL-C [13]. Both loci were significantly associated with LDL-C in the present study as well (Table 2), as previously reported [14]. In addition, the *APOE/APOC1* locus was weakly associated with HDL-C. We also confirmed the *ZNF259/BUD13* locus association with HDL-C and triglyceride levels as reported in genome-wide association studies, including the Framingham Heart Study [14]. *SCARB1* polymorphisms have been associated with HDL-C, triglycerides, and LDL-C in candidate-gene studies [15]–[22], but not in genome-wide association studies. In the current study, however, the *SCARB1* SNP was not associated with any of these lipids/lipoproteins.

### Analysis of top SNP effects on Lp-PLA_2_ activity controlling for lipids/lipoproteins

Because the top SNPs for Lp-PLA_2_ activity were in gene regions known to associate with lipoprotein levels, we next asked whether these SNPs remained significantly associated with Lp-PLA_2_ activity beyond their reported association with serum lipids/lipoproteins. For each SNP, we added LDL-C, HDL-C, or triglycerides as a covariate to the original regression model. The association between SNPs at the *CELSR2/PSRC1* locus and Lp-PLA_2_ activity was substantially reduced in a model accounting for their association with LDL-C, but not eliminated (Table 4). In contrast, the *APOE/APOC1* locus remained strongly associated with Lp-PLA_2_ activity, even after accounting for its association with LDL-C and HDL-C. Similarly, when modeling the association between Lp-PLA_2_ activity and the SNPs at the *ZNF259/BUD13* locus, including triglycerides or HDL-C as covariates did not appreciably reduce the association. *SCARB1* was not evaluated since the SNP showed no association with lipoproteins in this study. Similarly, we did not further evaluate the *PLA2G7* SNP, with respect to mass or activity, because it was not associated with any lipoproteins.

**Table 4:** Comparison of association of top activity SNPs in statistical models with and without serum lipid/lipoprotein levels, presented as regression coefficient (p value).

SNP	Model 1	Lipid/lipoprotein	Model 2
rs4420638 (*APOC1*)	8.0 (6×10^−24^)	LDL-C	5.6 (2×10^−15^)
rs4420638 (*APOC1*)	8.0 (6×10^−24^)	HDL-C	7.1 (2×10^−22^)
rs599839 (*PSRC1*)	−5.5 (3×10^−15^)	LDL-C	−2.6 (3×10^−5^)
rs12286037 (*ZNF259*)	6.4 (4×10^−8^)	HDL-C	4.6 (2×10^−5^)
rs12286037 (*ZNF259*)	6.4 (4×10^−8^)	Triglycerides	5.4 (3×10^−6^)
rs11820589 (*BUD13*)	6.2 (1×10^−7^)	HDL-C	4.6 (3×10^−5^)
rs11820589 (*BUD13*)	6.2 (1×10^−7^)	Triglycerides	5.3 (5×10^−6^)

Model 1: controlled for age, age^2^, sex, smoking, lipid-lowering medication, and cohort. Model 2: included all Model 1 covariates and the indicated serum lipid/lipoprotein.

### Analysis of top activity SNPs associated with apolipoprotein levels

Because our top loci all encompassed genes related to lipid metabolism, and yet their association with lipid or lipoprotein levels could not account for their association with Lp-PLA_2_ activity, we considered whether the effect of the SNPs was mediated through some other factor. Some of our top hits are in regions harboring apolipoprotein genes, so we examined the association of our top loci with levels of apolipoproteins A1, B, and E (measured only in the Offspring cohort). In addition, we expect Lp-PLA_2_ activity to correlate with apolipoprotein B levels. The *CELSR2/PSRC1* and *APOE/APOC1* SNPs were strongly associated with apolipoprotein B levels, and the *APOE/APOC1* SNP was also very strongly associated with apolipoprotein E levels (Table 2). None of our top SNPs were associated with apolipoprotein A1 levels. We were unable to further investigate the relationships between associated SNPs, apolipoprotein levels, and Lp-PLA_2_ activity because the apolipoproteins were measured at different exams than Lp-PLA_2_ activity.

## Discussion

To gain insight into genetic variants that control Lp-PLA_2_ activity and mass, we conducted a genome-wide association study using data from the Framingham Heart Study released through the SHARe Project. We identified different loci associated with each trait: four loci associated with Lp-PLA_2_ activity and one associated with Lp-PLA_2_ mass. Variants in the region of *PLA2G7*, encoding lipoprotein-associated phospholipase A2, were associated with Lp-PLA_2_ mass, the strongest association being with a common non-synonymous change, H92R. The four loci associated with Lp-PLA_2_ activity all harbor genes previously associated with lipoprotein levels and known to be involved in lipid metabolism. However, all of these loci remained significantly associated with Lp-PLA_2_ activity after accounting for their association with serum lipid and lipoprotein levels. Interestingly, a fifth activity locus, *PLA2G7*, emerges as significant only after controlling for mass.

Ours is not the first study to examine genetic variants associated with Lp-PLA_2_ activity and mass; however, it is the first to do so on a genome-wide level. A limited number of SNPs in *PLA2G7*, encoding lipoprotein-associated phospholipase A2, have been examined for association with Lp-PLA_2_ activity or mass. A common non-synonymous change, rs1051931 (A379V), was examined in several studies and found to be associated with activity [11], [23]–[25]. Abuzeid *et al.* [26] reported this same SNP associated with mass. According to Hapmap data, this SNP is not in linkage disequilibrium with our top SNP, rs1805017 (r^2^ = 0.07). A second common, non-synonymous European variant, rs1805018 (I198T), was associated with activity in Chinese population [27]. rs1805018 is not in linkage disequilibrium with either rs1051931 or rs1805017 (r^2^ = 0.02 for both). Neither the A379V nor I198T locus was associated with Lp-PLA_2_ activity (p = 1×10^−4^ and p = 0.8, respectively) or mass (p = 0.2 and p = 0.007) at a genome-wide level of significance in our study. Our top SNP associated with mass was also a common non-synonymous variant (H92R) in *PLA2G7*, and has not previously been associated with Lp-PLA_2_ activity or mass, but has been associated with coronary artery disease [28]. It is not clear whether the H92R change is the causal variant at this locus, or a marker of the causal variant. However, taken together with the other literature, our results suggest that the non-synonymous variants at this locus deserve further attention.

Our top locus for Lp-PLA_2_ activity, the *APOE/APOC1* region, has been previously reported by others. A candidate-gene study conducted by Drenos and colleagues examined the *APOE/APOC1* gene cluster for associations with multiple markers of cardiovascular disease and found a strong association with Lp-PLA_2_ activity as well as total cholesterol, LDL cholesterol, apolipoprotein B, and C-reactive protein [29]. Thus, our study provides further evidence for this candidate-region association and also implicates the *APOE/APOC1* gene cluster as the strongest locus associated with Lp-PLA_2_ activity on a genome-wide level. Interestingly, the rs4420638 SNP associated with Lp-PLA_2_ activity in our study has also been associated with LDL-C, Alzheimer's disease, and C-reactive protein in other genome-wide association studies [13], [30]–[32]. Each copy of the rs4420638 minor allele, G, was associated with increasing Lp-PLA_2_ Activity. Based on patterns of linkage disequilibrium with the *APOE e2/e3/e4* genotypes, we would expect that carriers of the *APOE e4* allele would have increased Lp-PLA_2_ activity compared to those with either the *e2* or *e3* alleles. In addition, we found a highly significant association between the rs4420638 SNP and apolipoprotein E levels. Whether these associations are causally related or represent pleiotropic effects of this locus remains to be determined. Early work on Lp-PLA_2_ suggested that it may be carried specifically on apolipoprotein E-containing HDL, and this may bear further investigation [33].

This is the first reported association between SNPs in the gene regions of *SCARB1*, *ZNF259/BUD13*, and *CELSR2/PSRC1* with Lp-PLA_2_ activity. Although the identity of each causal genetic variant and its target gene remains to be discovered, plausible candidates can be found at each locus. *SCARB1* encodes the scavenger receptor class B type 1 (SR-BI), and is well-recognized as an HDL receptor involved in reverse cholesterol transport. Despite the well-established role of this gene in lipid metabolism, to date genome-wide association studies, including those conducted with the Framingham Study, have not identified any genetic variants of *SCARB1* significantly associated with serum lipid levels (http://genome.gov/gwastudies/). However, recent studies have ascribed additional function to this receptor in regulating inflammatory responses. In particular, in a mouse model of sepsis, macrophages from SR-BI-null mice have been shown to produce significantly higher levels of inflammatory cytokines than those of wild type controls in response to LPS [34]. A recent study found that *SCARB1* rs10846744, associated with Lp-PLA_2_ activity in our study, was associated with subclinical atherosclerosis in the Multi-ethnic Study of Atherosclerosis [35]. The association was independent of lipoprotein levels and other metabolic risk factors. Given our findings, it is plausible that the association of *SCARB1* SNPs with cardiovascular disease in their study could be mediated through the inflammatory effects of *SCARB1*. The second locus associated with Lp-PLA_2_ activity in our study is in the *ZNF259/BUD13* region, which includes the apolipoprotein genes *APOA5*, *APOA4*, *APOC3*, and *APOA1*. This locus has been implicated in the control of serum triglyceride levels and increased coronary artery disease risk in a genome-wide association study [36]. *APOA1* encodes apolipoprotein A1, which resides on HDL, interacts with SR-BI, and has anti-inflammatory properties [37], [38] The last locus contains three genes, *SORT1*, *PSRC1* and *CELSR2*, which are relatively unexplored aside from their association with LDL cholesterol in numerous genome-wide association studies [13], [14], [36], [39]–[42]. Recent studies suggest that the associated SNP at this locus controls mRNA expression of the *SORT1* gene encoding sortilin 1 [13], [43].

Lp-PLA_2_ is noncovalently bound to human plasma LDL and HDL and the interaction with these lipoproteins largely determines its physiological activity [4], [44]. The majority of Lp-PLA_2_ in humans is bound to LDL, and subjects with elevated plasma LDL cholesterol levels also have high Lp-PLA_2_ activity [5]. Thus, the finding in our study that SNPs associated with serum lipoprotein levels (specifically those associated with LDL cholesterol levels) are also associated with Lp-PLA_2_ activity is not surprising. However, the associations between these SNPs and Lp-PLA_2_ activity could not be entirely accounted for by their associations with serum lipid/lipoprotein levels. Moreover, it is interesting to note that other known genetic determinants of LDL cholesterol levels, such as SNPs at the *APOB* locus are not significantly associated with Lp-PLA_2_ activity in our study. The *ZNF259/BUD13* locus is more strongly associated with triglycerides than LDL cholesterol in prior genome-wide association studies and in the present study. This result is consistent with the high Lp-PLA_2_ activity found associated with small dense LDL, since hypertriglyceridemia shifts the distribution of LDL toward smaller particle sizes [45]. Finally, *SCARB1* has not been associated with serum lipoprotein levels in any genome-wide association study or in the present study. Therefore, while lipid metabolism genes in general are rational candidates due to the physical association of Lp-PLA_2_ with LDL and HDL, the exact loci contributing to variation in Lp-PLA_2_ activity found in our study may provide further insight into the relationship between Lp-PLA_2_ activity and lipoproteins.

Our study was carried out in the population-based Framingham Heart Study, considered by some to be the gold standard for cardiovascular-disease-related biomarker discovery and validation [46]. Nonetheless, there are some caveats to note. First, quality-control procedures for the genotype data removed a large number of SNPs. It is possible that additional loci influencing Lp-PLA_2_ activity and mass exist, but were not evaluated in our analysis. Second, it is possible that the reported associations arise from confounding by lipoprotein subspecies or inflammatory indices not measured in the current study. We were able to explore this systematically for HDL-C, LDL-C, and triglycerides. However, within the Offspring cohort, Lp-PLA_2_ activity was measured at a different exam than apolipoprotein levels, limiting our ability to properly assess potential confounding by apolipoproteins. Finally, it would also have been informative to examine not just total Lp-PLA_2_ measures, but Lp-PLA_2_ by lipoprotein class and subclass, or indices of production by specific cell types, but these data were not available.

In summary, we report the first genome-wide analysis for SNPs associated with Lp-PLA_2_ activity and mass. For Lp-PLA_2_ activity, SNPs in the *APOE/APOC1* region, a region previously reported in a candidate-gene study, emerged as the top hit on a genome-wide scale. This same locus has been associated with apolipoprotein E levels, LDL cholesterol levels, inflammatory traits, and cardiovascular-disease outcomes. Future studies should be carried out to determine if this locus is pleiotropic, or if it controls a mediator related to both apolipoprotein E levels and Lp-PLA_2_ activity. Three other loci associated with lipoprotein phenotypes were also highly associated with Lp-PLA_2_ activity beyond their association with serum lipid levels. Our study also confirmed the importance of variants of the *PLA2G7* gene in determining Lp-PLA_2_ mass. Lp-PLA_2_ is an inflammatory marker that has been implicated in the progression of atherosclerosis, and clinical trials of Lp-PLA_2_ inhibitors are currently in progress. Knowledge of genetic variation that influences activity of Lp-PLA_2_ could have implications for therapeutic intervention in this pathway.

## Materials and Methods

### Ethics statement

This research was approved by the Duke University institutional review board and all clinical investigation was conducted according to the principles expressed in the Declaration of Helsinki.

### Study population

We conducted this research using data from the Framingham Heart Study (FHS), a population-based, longitudinal study of families living in the town of Framingham, Massachusetts collected over three-generations beginning in 1948. An overview of the FHS is provided at the dbGap website (http://www.ncbi.nlm.nih.gov/sites/entrez?db=gap) and detailed descriptions are available elsewhere [47], [48]. Briefly, the original study enrolled 5209 individuals, primarily Caucasian, and it later added the offspring of the original cohort (Offspring), and the grandchildren (Generation 3) of the original cohort.

All lipid-related traits were measured on fasting, plasma samples stored at −80°C. Lp-PLA_2_ activity was measured during the seventh exam of the Offspring cohort and first exam of the Third Generation cohort using a colorimetric method (diaDexus CAM Kit, Inc., San Francisco, CA). Apolipoprotein E concentrations were measured during the fifth Offspring exam using an immunochemical technique. Apolipoproteins A1 and B were measured at the fourth exam by noncompetitive ELISA with the use of affinity-purified polyclonal antibodies [49]. Triglycerides, total cholesterol, and HDL-C were measured using standard enzymatic methods, and LDL-C was calculated using the Friedewald formula [50] if triglycerides levels were less than 400 mg/dL. Smokers were defined as individuals who indicated that they smoked regularly within the past year of the exam.

### Genotype data and quality control

Genome-wide genotypes and detailed clinical data on subjects from all three generations have been made accessible to the research community through the SHARe project (SNP-Health Association Resource). The study protocol was approved by Duke University's Institutional Review Board and the Framingham SHARe Data Access Committee. The unfiltered genotype data contained 9215 individuals (all generations) genotyped for 549782 SNPs. This included 500568 SNPs from the Affymetrix 500K mapping array and 49214 SNPs from the Affymetrix 50K supplemental array (Affymetrix, Santa Clara, CA, USA). We used the toolset PLINK to perform quality control [51]. Individuals were excluded if genotyping rates were less than 97%. Markers were excluded if genotyping rates were less than 97%, minor allele frequencies were less than 0.05, or if Hardy-Weinberg p-values were less than 0.001. All SNP exclusions were made sequentially in the preceding order. Using this filtered data, we checked for Mendel errors using a 5% cutoff per family, and a 10% cutoff per SNP (as defined in PLINK), but none were detected. Individuals were also excluded if the predicted sex based on X-chromosome genotypes did not match the recorded sex. Pair wise identity-by-descent measures were calculated to detect replicated samples and unknown inter-familial relationships. We detected four sets of identical twins and randomly selected one member of each pair for the analytic sample. After quality controls, the remaining sample consisted of 8493 individuals genotyped on 360811 SNPs, attaining a genotyping rate of 99.5%. We also removed individuals who had missing values for any covariates.

### Statistical analysis

The primary analytic sample included 6668 individuals who had both genotypes and Lp-PLA_2_ measurements available. We tested SNPs for their association with Lp-PLA_2_ activity and mass in an additive genetic model. To account for relatedness, we used linear mixed models implemented in the kinship package of the R statistical language. This allowed fitting an individual random effect that was correlated according to the degree of relatedness within a family [52]. Regression models included the covariates age, age^2^, sex, smoking status, a variable indicating the use of cholesterol-lowering drugs, and a cohort variable corresponding to the generation within the FHS. Subsequent analyses also included serum lipoproteins in the regression model. We also tested for evidence that population stratification was inflating test statistics. To do this, we estimated the inflation factor by dividing the median of the observed χ^2^ statistics by the expected median in the absence of stratification (0.456) [53]. We defined genome-wide significance using a Bonferroni cutoff of 1.4×10−7, which corrects for 360811 tests. Following genome-wide analysis, we annotated results using the WGAViewer package, Ensembl, and the UCSC genome browser [54]–[56]. We generated plots using the ggplot2 package [57] and Haploview software [58].

## Supporting Information

Table S1 Analysis of SNPs associated with Lp-PLA_2_ activity.
(8.01 MB TXT)

Table S2 Association of SNPs with Lp-PLA_2_ mass.
(8.37 MB TXT)
